# Preregistered test of whether a virtual nose reduces cybersickness

**DOI:** 10.1186/s41235-024-00593-3

**Published:** 2024-10-29

**Authors:** Sai Ho Yip, Adrian K. T. Ng, Henry Y. K. Lau, Jeffrey A. Saunders

**Affiliations:** 1 University of Hong Kong, Pok Fu Lam, Hong Kong; 2 University of Bristol, Bristol, UK; 3 United Kingdom Atomic Energy Authority, Abingdon, UK

**Keywords:** Cybersickness, Simulator sickness, Visually-induced motion sickness, Virtual reality, Virtual nose, Rest frame hypothesis, FMS, SSQ

## Abstract

Recent findings suggest that adding a visual depiction of a nose to virtual reality displays (virtual nose) can reduce motion sickness. If so, this would be a simple intervention that could improve the experience of a variety of VR applications. However, only one peer-reviewed study has reported a benefit from a virtual nose, and the effect was observed in a single low-powered experiment. To further test the effectiveness of a virtual nose for mitigating motion sickness in VR, we performed a preregistered experiment with higher power and better control. Subjects were presented with simulated movement in a virtual environment using a head-mounted display, and the resulting motion sickness was measured using the Fast Motion Sickness Scale (FMS) and the Simulator Sickness Questionnaire (SSQ). Conditions with and without a virtual nose were tested in separate sessions on different days, and the mean habituation effects were removed analytically. Awareness of the manipulation was assessed with a funnel debriefing procedure. The sample size (*n* = 32) was chosen to have over 90% power to detect the estimated effect size based on previous data (*d*_*z*_ = 0.6). We found no significant difference between motion sickness in conditions with and without the virtual nose. The estimated effect size was close to zero, *d*_z_ = − 0.02, with a 95% credible interval [− 0.37, 0.33]. Results from a Bayesian analysis imply that any benefit from a virtual nose is unlikely to be more than a 26% reduction in FMS scores, and any cost is unlikely to be more than a 23% increase. Our results do not support the hypothesis that a virtual nose is a general and effective way to relieve motion sickness in virtual reality.

**Supplementary Information:**

The online version contains supplementary material available at 10.1186/s41235-024-00593-3.

## Introduction

Virtual Reality (VR) enables its users to be immersed in a simulated environment, typically with the ability to navigate around the virtual world. With the release of commercial VR head-mounted displays (HMD), the technology has become more accessible to the general population. Researchers and developers have utilized VR for numerous recreational, vocational training, and rehabilitation purposes (Hamad & Jia, 2022; Palkova & Hatzilygeroudis, 2019; Tokgöz et al., 2022).

However, cybersickness remains a persistent obstacle to the mainstream adaptation of VR (Chandra et al., 2022). Cybersickness refers to a series of motion sickness symptoms elicited by VR exposure (Arcioni et al., 2019; McCauley & Sharkey, 1992; Saredakis et al., 2020). The symptoms include, but are not limited to disorientation, oculomotor discomfort, eyestrain, and nausea (Caserman et al., 2021; Servotte et al., 2020). This is sometimes referred to as “Simulator Sickness”, but others have argued that simulator sickness is a different strain of motion sickness (Stanney et al., 1997). Cybersickness can also be considered a specific case of visually-induced motion sickness (VIMS), because a likely cause of the sickness is conflicting visual cues (Keshavarz & Golding, 2022).

While susceptibility to cybersickness tends to vary between individuals, Lawson (2015) estimated that around 61–80% of the population will experience some form of cybersickness symptoms. Some researchers have cautioned that if no effective countermeasure against cybersickness is identified, similar to other stereopsis techniques (e.g., Nvidia 3D Vision), the interest in VR will eventually come to an end (Wienrich et al., 2022).

### Theories of cybersickness

The prevalence of cybersickness, as well as the significant threat it poses to VR advancements, has motivated research on the causes and possible mitigations of cybersickness (Brooks et al., 2010). Multiple theoretical frameworks have been proposed to explain the course of cybersickness, such as the sensory conflict theory, the postural instability theory, and the rest-frame hypothesis (Prothero, 1998; Reason, 1978; Riccio & Stoffregen, 1991).

The sensory conflict theory posits that cybersickness is the result of a mismatch between the motion signals from visual and non-visual (mainly vestibular) systems (Chang et al., 2020; Reason, 1978). Virtual navigation (i.e., moving an in-game avatar with a controller) creates an unnatural situation where visual motion cues are not accompanied by vestibular and proprioceptive cues that are normally present during movement. Conflicts between visual and vestibular cues can also arise due to lags between detecting real head movement and updating virtual displays (Palmisano et al., 2020). This atypical combination of sensory input is theorized to be misinterpreted as a sign of intoxication, leading to nauseogenic responses in an attempt to expel the toxicity (Treisman, 1977). It should be noted, however, that the sensory conflict is nauseogenic only if the experienced conflict is novel to the users (Reason, 1978). With habituation, susceptible individuals could adapt to their internal expectations, eventually experiencing little to no sickness from the same level of sensory conflict.

Although widely accepted by the research community, the sensory conflict theory offers limited implications for the prevention of cybersickness. Although habituation can be an effective countermeasure, users may not be willing to undergo more VR exposures if the initial experience is highly discomforting. Another possible solution would be to introduce non-visual motion feedback. For example, some studies have observed reduced sickness when vibrations were added to passive simulations of movement (D'Amour et al., 2017; Lucas et al., 2020). However, these studies do not provide clear evidence that the benefit was due to sensory congruence, and adding non-visual motion signals may not be feasible for many applications.

The postural instability theory posits that cybersickness is the result of unstable postural control (Riccio & Stoffregen, 1991). It predicts that the degree of standing postural instability corresponds to the cybersickness severity. The more unstable one’s postural is before the exposure, the more likely the individual will experience cybersickness. With experience in a virtual environment, people could potentially learn to stabilize posture despite the sensory conflicts, analogous to adapting “sea legs” in response to experience on a boat. However, empirical support for this theory appears to be mixed (Palmisano et al., 2020), and it provides little insight into the specific properties of virtual environments that contribute to cybersickness.

### Rest-frame hypothesis

The rest frame hypothesis suggests that cybersickness depends on the compatibility between experienced self-motion and the selected rest frame (Prothero, 1998; Somrak et al., 2021). In any instance, our nervous system will extract various reference frames from the scene, to gauge the orientation, location, and motion of objects (Heutink et al., 2019). A rest frame, in particular, is the reference frame that the nervous system selects and assumes to be stationary at any given moment. By comparing the selected rest frame and objects’ reference frames, one can derive whether an object is stationary or not. For instance, drivers may select the road as the current rest frame, and infer self-motion when the “static” rest frame (i.e., road) appears to be “moving”.

If the observer fails to select a reliable rest frame, motion sickness may arise from the conflict between the selected rest frame and visual cues (Somrak et al., 2021). If VR users are unable to select a rest frame that can accurately reflect the motion of its relative objects, cybersickness may occur from the confusion. Imagine a scenario where a passenger wakes up from a nap, while having their window view obscured by a passing bus. In this instance, a reliable rest frame (i.e., the road) is rendered unavailable momentarily, and the nervous system might mistake the moving bus for a rest frame. As the self-motion inferred from the current rest frame (in which “moving” rest frame indicates self-motion) conflicts with reality (in which the vehicle is stationary), the passenger would experience a brief sense of vertigo and disorientation.

While sharing some similarities with the sensory conflict theory, the rest frame hypothesis proposes that the internal conflict could be reduced even if there remain conflicts in the sensory input. Cybersickness can be alleviated by adding a stable, consistent, and readily available rest frame into the scene. Prothero et al. (1999) found that by making the laboratory wall visible during simulation, subjects experienced significantly less sickness. Implementing an independent visual background into the virtual world, such as an earth-fixed grid or simulated clouds, has also been found to reduce motion sickness (Duh et al., 2001a, 2001b). These early studies used virtual reality presented on project screens. A more recent study using an HMD found that adding a player-fixed black metal net allowed subjects to perform virtual navigation longer without significant discomfort (Cao et al., 2018). In all of these studies, the additional visual reference frame was fixed relative to the body and the physical environment. The visual reference frame could reinforce the body-based reference frame, ensuring the congruence between the selected rest frame and actual self-motion regardless of the conflicting visual information about self-motion.

Although these countermeasures proved to be effective at reducing motion sickness, they might often interfere with the goal of applications. Implementations such as a grid may appear unnatural to the users and impair their sense of presence. In-game background objects, like the virtual clouds, might be unavailable during in-door navigation. Hence, there is a need to identify an always-available, natural-looking object to serve as the in-game rest frame.

### Virtual nose as a rest frame?

Recent studies suggest that a virtual nose could serve as an always accessible yet minimally intrusive rest frame to combat cybersickness. Whittinghill et al. (2015) first reported a virtual nose effect in an unpublished study. They found that adding a virtual nose allowed subjects to endure VR experiences longer before reporting cybersickness. Another study by Wienrich et al. (2022) attempted to replicate the virtual nose effect, and also verify whether awareness is needed for the nose to be effective. Their results from the main experiment suggest that the virtual nose significantly reduced cybersickness, while finding no evidence for the hypothesized effect of awareness.

The authors attributed the observed effect to the rest frame hypothesis, suggesting that the virtual nose provided the subjects with a reliable and valid rest frame reflective of real-world motion (Whittinghill et al., 2015; Wienrich et al., 2022). During simulated movements in VR, the virtual nose will always appear to be stationary, independent of the other self-motion cues provided by the HMD. If the stationary virtual nose were selected as the rest frame, it could potentially reinforce the physical cues that indicate a lack of movement. By this explanation, cybersickness would be reduced through the minimization of conflict between the selected rest frame and actual self-motion.

**Fig. 1:**
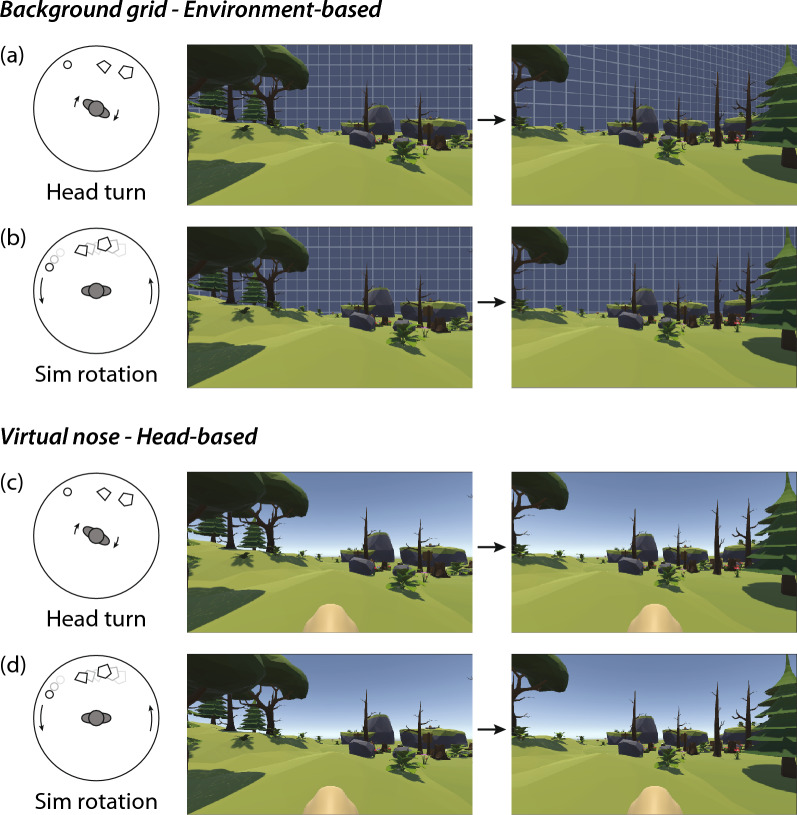
Illustration of the difference between an environment-based and head-based visual reference frame. The top rows show the case of a background grid that remains fixed relative to the external environment. It provides a visual reference frame that remains consistent with feedback from physical sensory cues, and can distinguish between rotational motion caused by head movements (**a**) and simulated rotations (**b**). The bottom rows show the case of a virtual nose. Because the virtual nose moves with the head, it would not help to distinguish head rotations (**c**) and simulated rotations (**d**). Unlike the fixed grid, the virtual nose could not serve as a reference frame for resolving sensory conflicts during simulated rotation

Although it may seem intuitive that the virtual nose can act as a reliable rest frame, it does not provide the same information as an environment-based visual frame. Figure 1 illustrates the difference between an environment-based visual reference frame and a head-based virtual reference frame. Unlike the virtual reference frames in studies discussed in the previous section, a virtual nose does not provide a stable rest frame that is fixed relative to the external physical environment. In particular, the virtual nose would not help to resolve conflicting information about self-rotation. A visual reference, such as a background grid, that is fixed relative to the external environment will rotate relative to the viewer in response to real head movements (Fig. 1a) but not in response to the simulated rotation of the avatar (Fig. 1b). Because the feedback distinguishes between rotation caused by head movements and rotation caused by simulated rotation, it could help reinforce the physical reference frame. In contrast, a head-fixed visual reference frame, like the virtual nose (Fig. 1c and d), provides no information about whether a rotation is caused by head movements or virtual rotation. To the extent that conflicting information about self-rotation contributes to motion sickness in VR, a virtual nose would not be helpful. While a virtual nose shares some characteristics of an environment-based reference frame, it does not provide a stable rest frame that could resolve the conflict caused by simulated rotation.

A recent study by Hemmerich et al. (2020) compared the effects of adding environment-based or head-based reference frames to virtual displays during simulated motion. They found that adding a line indicating the horizon of the physical environment reduced motion sickness, whereas a horizontal line yoked to the head orientation provided no benefit. These results provide further reason to doubt the rest frame explanation for a virtual nose effect.

### Other explanations for the virtual nose effect

Alternatively, instead of serving as a rest frame, the virtual nose might reduce cybersickness by enhancing the sense of presence. Presence is defined as the sense of “being inside” a virtual environment (Weber et al., 2021). While the relationship between presence and cybersickness has been widely debated, studies have shown that allowing users to see their body parts enhances the sense of presence, and also reduces the self-reported cybersickness scores (Weech et al., 2019). It is possible that the virtual nose facilitated the sense of presence. As the users become more immersed and engaged with the virtual experience, they begin to downplay any experienced cybersickness symptoms, leading to a lower self-reported cybersickness severity.

Another possibility is that the virtual nose prevents cybersickness by reducing the stereoscopic field of view. Palmisano et al. (2019) found that monocular viewing of a virtual environment with an HMD produced less cybersickness than binocular viewing, suggesting that stereopsis contributes to cybersickness. The presence of a virtual nose, regardless of whether it is a 2D or 3D model, will always introduce a small monocular field in both eyes. As such, it is possible that the larger monocular visual field resulting from the virtual nose protects the users from experiencing cybersickness.

### Need for replication

In addition to the unclear theoretical basis, several crucial factors call for a replication of the virtual nose effect.

Currently, there is only very limited empirical support for the proposed effect. The first study that reported the sickness-alleviating effect of the virtual nose, Whittinghill et al. (2015), remains unpublished. To our knowledge, Wienrich et al. (2022) is the only peer-reviewed published study that tested the effect of a virtual nose on cybersickness.

Furthermore, Wienrich et al. (2022) did not find consistent support for the virtual nose effect across their two experiments. The main experiment found that the virtual nose conditions had significantly less cybersickness as measured by the nausea scale of simulator sickness questionnaire (SSQ), and trends in the same direction for the SSQ-total and the fast motion sickness (FMS) scale. However, their pilot study using a similar method did not find a reduction in cybersickness in the nose-present conditions, and the trend was in the opposite direction. Also, the effect of the virtual nose on SSQ-nausea in the main experiment was only significant if a one-tailed test was used (*p* = 0.09 for a two-tailed test), which the authors did not justify. The inconsistent findings across experiments and measures suggest that the virtual nose effect might not be robust.

Another issue is that the experiment by Wienrich et al. (2022) was highly underpowered. For the nausea scale of the SSQ, which showed the strongest effect of the virtual nose, the effect size of the difference reported by Wienrich et al. (2022) was *d* = 0.52. The sample sizes used by Wienrich et al. (2022), *n* = 20 for the nose-present groups, and *n* = 10 for the nose-absent group, provided only 26% power to detect an effect with size *d* = 0.52. We also computed the effect size from their FMS results and found an estimated Cohen’s *d* = 0.66. Even for this effect size, the study was underpowered (38%). As underpowered studies can lead to an inflated effect size (Rochefort-Maranda, 2021), it is necessary to perform a well-powered follow-up experiment to accurately assess the true virtual nose effectiveness.

### Present study

This study aims to provide a stronger and more conclusive test of whether adding a virtual nose can reduce motion sickness in VR.

We conducted a preregistered experiment with higher power than Wienrich et al. (2022). We used a within-subjects design, which is more sensitive, and a larger sample size (*n* = 32) was chosen based on a power analysis of the expected effect. Subjects performed a virtual navigation task that required them to identify multiple targets hidden within a virtual forest. Two experimental conditions were created, either with the virtual nose being present or absent (control). The virtual task is expected to induce cybersickness, and we measured their cybersickness using two self-administered scales. It is hypothesized that compared to the control group, the presence of a virtual nose should significantly reduce the severity of cybersickness experienced in the virtual navigation task.

For exploratory analysis, we also measured self-reported spatial presence. If an effect of the virtual nose was caused by an enhanced sense of presence, then we would expect spatial presence to covary with cybersickness.

## Methods

### Openness and transparency

The experiment presented here was preregistered on OSF: https://osf.io/zcaes. The only deviation from the preregistered plan was additional analyses comparing the virtual nose conditions after controlling for an order effect. Removing the order effect provides a more sensitive measure of the possible effect size, but did not affect the qualitative findings. All data and analyses are available in a public repository: https://osf.io/dyma4.

### Participants

Thirty-two subjects, with an age range from 18 to 36, were recruited from the student population at the University of Hong Kong. All subjects had normal or corrected-to-normal vision, and had no known history of vestibular or neurological functioning impairment. We limited the age to 40 because some evidence suggests that older people are less susceptible to cybersickness (Saredakis et al., 2020). We recruited equal numbers of men and women to allow exploratory analyses of possible sex differences (e.g., Tian et al., 2022). Subjects were either paid $100 HKD/hour or course credits as compensation.

The sample size was chosen to have high power to detect a difference with effect size *d*_*z*_ = 0.60. We used the results of Wienrich et al. (2022) to estimate the effect size using the FMS scale, which is our primary measure. When combining their virtual nose conditions, the difference between the pre-post exposure FMS scores was 1.95. The average standard deviation of FMS ratings across subjects was 2.95. The variability of differences in a within-subject design is typically smaller than the variability across subjects, so we used 2.95 as a conservative estimate of the variance. Based on these estimates, the effect size would be expected to be *d*_*z*_ > 0.66. We targeted a slightly smaller effect size of *d*_*z*_ = 0.6. Our sample size of *n* = 32 provides 90.8% power to detect an effect size of *d*_*z*_ = 0.6 at an alpha level of 0.05. This power was computed for the case of a standard two-sided paired t-test using the non-central t-distribution under the assumption of normally distributed noise.

### Apparatus and stimuli

The experiment consisted of a virtual-navigation task. Subjects were asked to move through a simulated environment with scattered targets.

Three virtual environments were constructed for the virtual navigation task—two main environments and a training room. The main environments resembled a cartoon-like forest, populated by various trees, hills, campsites, ruins, and bushes. The only difference between the two environments was the specific arrangement of features and targets. The virtual scene was coded with Unity version 2019.4.8f1 and presented with an HTC Vive head-mounted display (HMD). The environment was rendered at a resolution of 2160 × 1200 (1080 × 1200 per eye) at a 90 Hz refresh rate. The viewing interpupillary distance (IPD) was set to 62.5 mm. Subjects translated virtual movements and turnings with the HTC Vive controller touchpad. The upper and lower parts of the touchpad were used to input forward/backward movements, while the left and right sides were used to control simulated rotation.

The rate of virtual translation and rotation was chosen so that subjects would be expected to show motion sickness symptoms within 4–6 min. The forward/backward speed was 6 m/s, and the rotation rate was 40°/s. We used similar parameters in our previous study (Yip & Saunders, 2023).

**Fig. 2:**
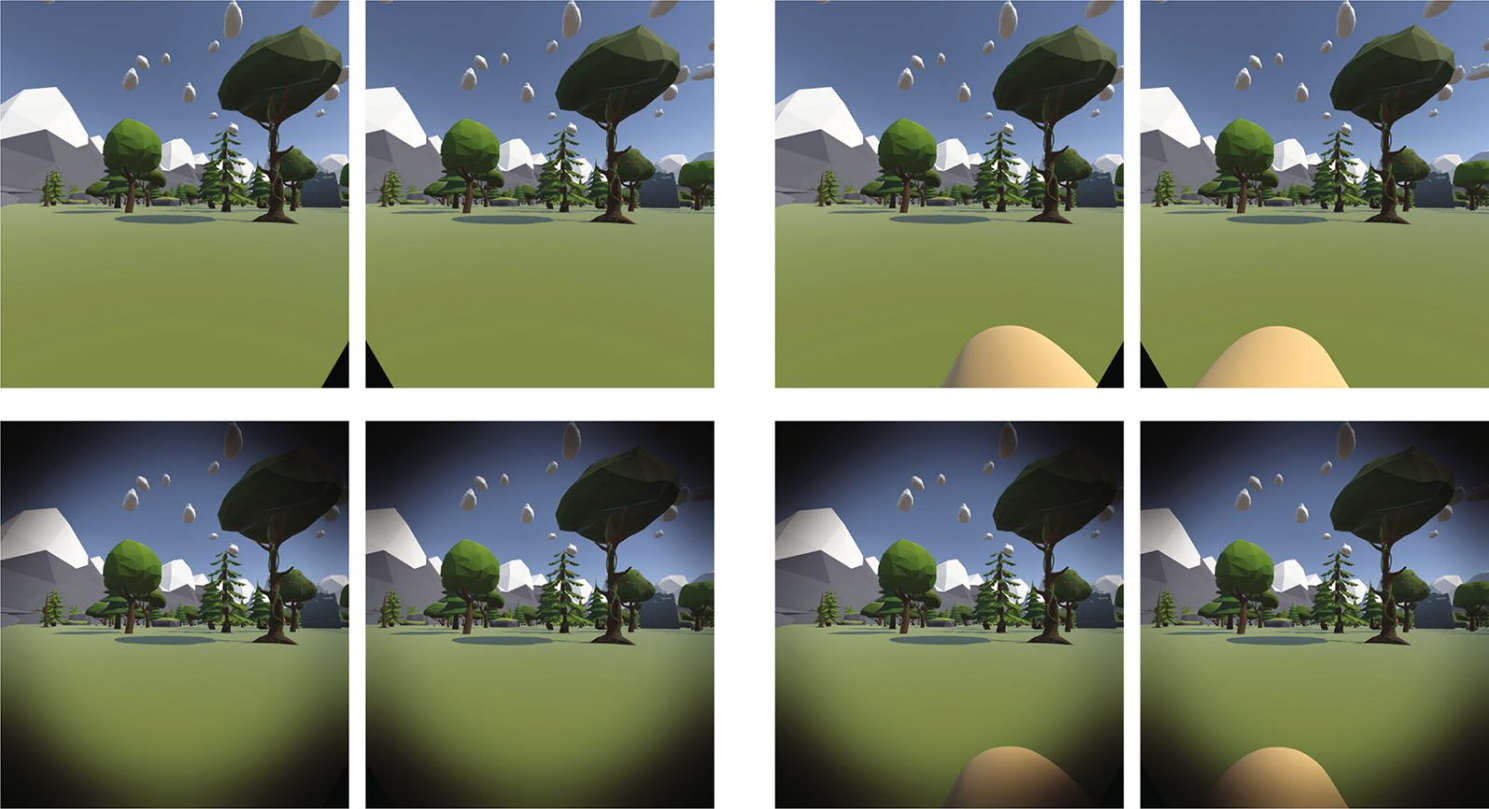
Binocular screenshots of the nose absent (left) and nose present (right) conditions. The top row shows the full binocular images. The bottom row shows images with added vignettes that approximate the reduced visibility of the images when viewed through the HMD

Two virtual nose conditions (nose-present VS nose-absent) were tested in a within-subject design, administered on two separate days. For the nose-present condition (Fig. 2), a 3D virtual nose was placed in front of the simulated cameras and moved with the subject’s head. The virtual nose was positioned to be visible in the bottom middle areas of the HMD displays. In order to be visible, the virtual nose was higher and farther from the face than the subjects’ actual nose. The virtual nose was rendered as an opaque object. It occluded different regions of the visual field in each eye, resulting in a semi-transparent appearance (like a real nose).

Subjects were instructed to “move” and collect target objects scattered in the environment. The order of the virtual nose conditions and the forest layouts was fully counterbalanced.

### Measurements

#### Fast motion sickness scale (FMS)

The Fast Motion Sickness Scale (FMS) was used as our primary measure. It is a single-item measure that monitors the course of cybersickness (Keshavarz & Hecht, 2011). For every two minutes of exposure, subjects were prompted to verbally rate their cybersickness severity on the FMS scale, ranging from 0 (no sickness at all) to 20 (frank sickness). When reporting their FMS score, subjects focused on current nausea, general discomfort, and stomach problems, while ignoring other irrelevant symptoms (i.e., boredom, nervousness).

Because the procedure was stopped when subjects experienced more than mild discomfort, we expected the exposure duration to vary across subjects and conditions. If subjects did not complete the full 12 min of exposure in both conditions, we analyzed the FMS scores from the latest time that was measured in both conditions. For example, if a subject completed 8 min in the nose present condition and 6 min in the nose absent condition, we used the FMS scores at 6 min from each condition. This ensures that the analyzed FMS scores reflect equal amounts of exposure in both conditions. In the experiment, the exposure duration varied between 4 and 12 min, with most subjects (29/32) completing at least 6 min in both conditions.

For analyses of individual differences, we used the rate of increase in FMS scores rather than the latest FMS scores available from each subject. Using the latest FMS scores would compress individual differences in cybersickness because more susceptible individuals would stop after shorter durations. This is not a problem for within-subjects comparisons, but is disadvantageous for analysis of individual differences. To deal with the issue of different exposure times, we analyzed the rate of increase in FMS scores as a function of exposure time. For each subject and condition, we performed a linear regression to the set of FMS scores at different exposure times. The slope, which is the rate of increase in FMS per minute, was used as a measure of an individual's rate of cybersickness.

#### Simulator sickness questionnaire (SSQ)

As a secondary measure, we also administered the Simulator Sickness Questionnaire (SSQ) both before and after the VR exposure. The SSQ is a commonly used self-reporting measure consisting of 16 items (Kennedy et al., 1993). Each item measured the severity of a particular motion sickness symptom (e.g., nausea) on a scale of 0–3. The SSQ can generate a total score, nausea, oculomotor disturbances, and disorientation subscale scores. All raw SSQ scores were calculated using the weighting procedure suggested by Kennedy et al. (1993).

We applied a log_10_(SSQ/10 + 1) transformation to the raw SSQ scores to reduce the skewness of the distribution. This transformation was chosen based on analyses of previous data from our lab. To compensate for any pre-existing motion sickness symptoms, we subtracted the pre-exposure log-transformed SSQ scores from the post-exposure log-transformed SSQ scores. Statistical analyses on the SSQ data were performed on the transformed and normalized scores.

#### Igroup presence questionnaire (IPQ)

To measure the sense of presence, subjects completed the Igroup Presence Questionnaire (IPQ) at the end of the navigation task. The IPQ is a 14-item subjective questionnaire that assesses the users’ level of spatial presence, involvement, and perceived realism of a VR experience (Schubert, 2003; Schubert et al., 2001). The IPQ consists of one general item and three different subscales—the sense of physical presence in the virtual environment (spatial presence—SP), the level of attention and involvement devoted to the VR experience (involvement—INV), and the degree of realism perceived from the simulated environment (experienced realism—REAL). All responses were rated on a 7-point scale (0–6) and were presented as described in the original work (Schwind et al., 2019; Tao & Archambault, 2016).

We analyzed the results from the three subscales rather than the total score because the components were not equally relevant to our experiment. If a virtual nose increased the sense of being in the environment, this change would be most reflected in the spatial presence component (e.g., “Somehow I felt that the virtual world surrounded me.”). It is not clear how the virtual nose would influence involvement with the task, so the involvement component (e.g., “I still paid attention to the real environment.”) is less relevant. The experience realism component (e.g., “How real did the virtual world seem to you?”) is unlikely to be useful because our environment was not designed to be photorealistic, and users could potentially have a strong sense of physical presence despite the cartoon nature of the stimulated world.

#### Navigation performance

To assess navigation performance, we recorded the trajectories during navigation and computed three measures: (1) average speed of translational movements, (2) average speed of simulated rotations, and (3) proportion of time without simulated movement. For the measure of average translational speed, we computed the change in player position in the horizontal plane across each pair of frames and averaged over the navigation period. Similarly, the average speed of simulated rotation was computed using the angular difference in player orientation (independent of physical head movement) across each pair of frames and averaging over the navigation period. Time without simulated movement was computed by summing all recorded frames during which the subject was not pressing the controller touchpad. Physical head rotations were not treated as simulated movements.

## Procedure

This study was a two-day experiment. Each experimental session tested a specific virtual nose (nose-present VS nose-absent) condition. At the start of a session, we administered the pre-exposure SSQ and FMS. The experimenter then assisted the subjects to put on the HMD and initiated the virtual navigation task. The virtual navigation was performed for 12 min or until subjects stopped due to discomfort, and subjects reported their FMS score every 2 min. At the end of the virtual navigation, the subjects completed the post-exposure SSQ and the IPQ.

During the navigation task, subjects moved through the virtual forest and collected as many treasure chests as possible within 12 min, or until they experienced more than moderate discomfort. The chests were collected by moving in close proximity to them, triggering an opening animation in the process. Every 2 min, subjects were prompted to verbally report their current FMS score. If they reported an FMS score ≥ 14, we checked whether they wanted to continue, and immediately terminated the navigation task if they did not.

The 12-min maximum duration for the navigation task was chosen based on previous results using a similar task. In Experiment 1 of Yip and Saunders (2023), most subjects reported mild motion sickness symptoms within 4 min of exposure, and more than half of the subjects showed moderate symptoms after 8 min of exposure. Based on these findings, we expected that 12 min of navigation would be sufficient to elicit measurable symptoms from almost all subjects, and that most subjects would stop due to discomfort after 6–10 min.

Prior to the main navigation task, we asked the subjects to perform a short practice block to familiarize themselves with the controls and the task. The virtual practice environment was a grid-textured room with four treasure chests. Subjects were required to collect all four treasure chests before proceeding to the forest.

At the end of the experiment, we conducted a funnel debriefing to assess the subjects’ awareness of the manipulation and expected results. We first asked them: “What do you think the purpose of the experiment was?”. We then asked them if they noticed anything different about the displays in the two sessions. If they did not spontaneously identify the virtual nose as a difference, we explained that a virtual nose was present. We then asked them to identify which session had the virtual nose. Finally, we asked them to guess the direction of the hypothesized effect: whether the virtual nose would be expected to increase or reduce cybersickness.

## Results

### The effect of virtual nose on cybersickness

We performed paired-sample t-tests (two-tailed) to compare the mean cybersickness scores from the nose-present and nose-absent conditions. In accordance with our preregistered plan, analyses of FMS ratings used raw scores, and analyses of SSQ ratings used the log_10_(SSQ/10 + 1) transformed SSQ scores.

**Fig. 3:**
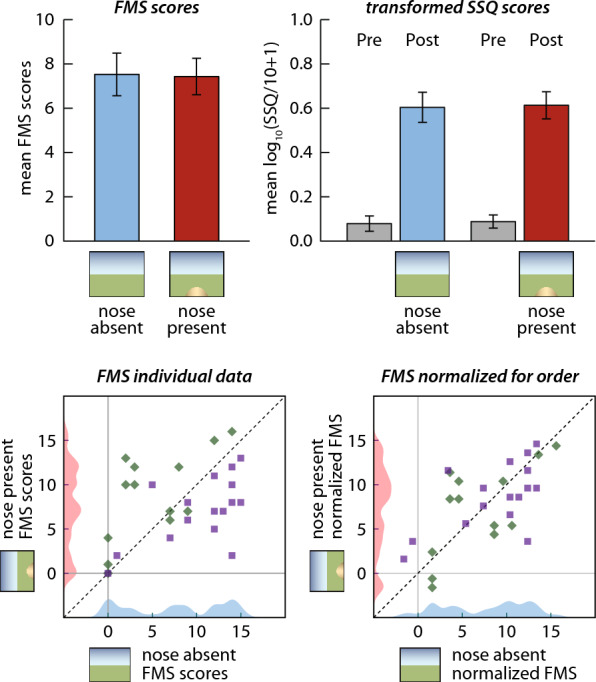
Top: Mean cybersickness averaged across subjects in the conditions with a virtual nose (red) and without a virtual nose (blue), as measured by FMS ratings (left) and SSQ-Total (right). The SSQ scores were transformed by log10(SSQ/10+1) for analysis. Error bars show ±1 standard error. Bottom: Scatter plots showing individual FMS results from nose absent and nose present conditions. The left graph plots raw FMS scores and the right graph plots FMS scores after normalizing for the mean order effect. Squares plot subjects who performed the nose absent condition first and diamonds plot subjects who performed nose present first. Density plots are shown along the axes

Figure 3 plots the mean FMS and transformed SSQ scores from the two virtual nose conditions. For our primary cybersickness measure, no significant difference in the FMS mean score was observed between the nose-present condition (*M* = 7.438, *SD* = 4.655) and nose-absent condition (*M* = 7.531, *SD* = 5.477), *t*(31) = − 0.105, *p* = 0.917, *d*_*z*_ = − 0.019). Null findings were also observed from our secondary measure, the transformed normalized SSQ scores. The mean change in log_10_(SSQ-Total/10 + 1) was statistically the same between the nose-present (*M* = 0.525, SD = 0.601) and nose-absent condition (*M* = 0.526, *SD* = 0.558), *t*(31) = − 0.009, *p* = 0.993, *d*_*z*_ = − 0.002. No significant difference between the two virtual nose conditions was observed from nausea (*t*(31) = 0.662, *p* = 0.513, *d*_*z*_ = 0.117), oculomotor disturbances (*t*(31) = − 1.054, *p* = 0.300, *d*_*z*_ = − 0.186), and disorientation (*t*(31) = 0.440, *p* = 0.663, *d*_*z*_ = 0.078) SSQ subscales either. Altogether, despite a sufficiently powered sample, we were unable to replicate the sickness-reducing effect of the virtual nose on cybersickness reported by the previous studies.

### Exploratory analysis: controlling the order effect

We observed that there was an overall difference between the mean cybersickness in the first and second experimental sessions. Because the design was counterbalanced, the order effect does not affect the mean difference. However, the order effect would still contribute noise to the analysis. As such, we performed an additional analysis with the order as a factor to test whether the order effect obscured an effect of the virtual nose. This additional analysis was not part of the preregistered plan.

We examined the difference in FMS scores between nose conditions using a 2 × 2 ANOVA, with the condition experienced first (nose-first vs. control-first) as the additional between-subjects factor. We found that there was a significant order effect, in which subjects tended to report higher FMS scores in their first session, *F*(1, 30) = 21.660, *p* < 0.001, *η*^*2*^_*p*_ = 0.419. Despite controlling the order effect, we still failed to detect any significant difference in the mean FMS scores between the nose conditions, *F*(1, 30) = 0.018, *p* = 0.893, *η*^*2*^_*p*_ = 0.001.

Similarly, our secondary measure of cybersickness, change in transformed normalized SSQ, showed no effect of the virtual nose even after accounting for the order effect. The SSQ scores did not show a significant difference between first and second sessions, *F*(1, 30) = 3.235, *p* = 0.082, *η*^*2*^_*p*_ = 0.097, but the trend was in the same direction as the order effect in FMS scores. When order was introduced as an additional variably in the analysis of SSQ scores, there remained no main effect of the virtual nose, *F*(1, 30) < 0.001, *p* = 0.993, *η*^*2*^_*p*_ < 0.001.

Consistent with previous observations, we found evidence that cybersickness was reduced after previous exposure to the VR conditions. However, there remained no evidence that the virtual nose reduced cybersickness even after accounting for the order effect.

### Bayesian analyses on the virtual nose effect

We conducted Bayesian analyses to compute credible intervals for the differences in cybersickness between the virtual nose conditions. This allowed us to estimate the true effect size range of the virtual nose at a specified confidence level (i.e., 95%).

For each cybersickness measure, we first estimated the posterior distribution for differences using the Bayes theorem, then computed the 95% highest density intervals (HDIs) of credible values. We assumed the noise to be normally distributed. For the unknown parameters in our analyses, we utilized non-informative priors: a uniform distribution for the mean difference, and a Jeffreys prior for the variance. The posterior distribution for mean differences was computed by numerically integrating over the variance parameter: P(m|{xi}) ~ ∫ P({xi}|m,v) (1/v) dv. Using the estimated posterior, we determined the smallest interval containing 95% of the posterior probability (i.e., 95% HDI). For the FMS, the HDI values correspond to the original raw scale. For the SSQ, we computed HDIs using log transformed scores and then converted the HDIs back to the original scale.

The results suggest that any reduction in cybersickness caused by the virtual nose was relatively small. The 95% HDI for the difference in mean FMS between the virtual nose conditions was [− 1.92, 1.72]. Relative to the mean FMS score in the nose-absent condition (7.53), the lower bound would be a 25.6% reduction in cybersickness and the upper bound be a 23.0% increase. For the SSQ data, the 95% HDI for the differences in log_10_(SSQ-Total/10 + 1) between the two conditions was [− 0.11, 0.11]. We then converted these values into the differences in raw SSQ total scores using the mean post-exposure SSQ in the nose-absent condition (47.2) as the baseline. The resulting 95% HDI for untransformed SSQ scores was [34.8, 62.9]. The lower bound for the difference in SSQ scores would correspond to a 26.2% reduction in cybersickness in the presence of a virtual nose, and the upper bound would correspond to a 33% increase. Note that the lower bound is similar to the bound determined from FMS results. If the virtual nose provides a benefit in our conditions that was not detected, our results indicate that the effect is unlikely to be more than a 26% reduction.

The bounds for the possible effect are smaller when the order effect is controlled. We repeated our Bayesian analysis of FMS results using the means after controlling for the order effect. This additional analysis was not in our preregistered plan. Using the normalized means, the 95% HDI for the difference in FMS across the nose conditions was [− 1.48, 1.29]. Relative to the nose-absent condition mean FMS score, the lower bound indicates a 19.8% reduction in cybersickness, and the upper bound would be a 17.3% increase. When the order effect is statistically controlled, our results imply that any beneficial effect of the virtual nose is unlikely to be more than a 19.8% reduction in sickness.

We also computed 95% HDIs for the effect size of a virtual nose effect on FMS scores. To estimate the posterior for effect size, we assumed a uniform prior. The 95% HDI of the effect size for a difference between FMS scores in the nose present and absent conditions was [− 0.365, 0.328]. This implies that any benefit from the virtual nose has a small effect size, with | *d*_*z*_ |< 0.37.

### Awareness of the virtual nose

If subjects noticed the virtual nose manipulation and had an expectation about the hypothesized effect, this could potentially cause a placebo effect. We analyzed the debriefing results to assess the subjects' awareness of the manipulation and tested for a possible interaction between awareness and the effect of a virtual nose.

When asked to report the purpose of the experiment, only 6 subjects (18.75%) correctly responded that the experiment was testing the effect of the virtual nose. Furthermore, among these subjects, only 4 were able to both accurately recall which session had the virtual nose and guess the direction of the hypothesis. This revealed that at most 12.5% of our subjects were fully aware of the purpose and expected effect.

We also considered the possibility that subjects had some partial awareness of the manipulation. Subjects who could identify the virtual nose manipulation and expected effect when prompted could potentially be biased even if they did not accurately report the purpose of the experiment. When prompted that the experiment involved the virtual nose, 71.88% (*n* = 23) were able to correctly report which session had the virtual nose, and 46.88% (*n* = 15) accurately guessed the direction of our hypothesis. In total, 40.63% of our subjects (*n* = 13) who showed some evidence that they were aware of the manipulation and expectations.

We performed an additional analysis to test whether there was an interaction between partial awareness and the effect of the virtual nose. We used partial awareness because it is a conservative estimate of the subset of subjects that were susceptible to bias. The FMS and transformed SSQ results were analyzed with a 2 × 2 ANOVA using partial awareness as a between-subject variable in addition to the virtual nose condition. We found no significant interaction for either FMS scores, *F*(1, 30) = 0.038, *p* = 0.847, *η*^*2*^_*p*_ = 0.001, or transformed SSQ scores, *F*(1, 30) = 0.054, *p* = 0.818, *η*^*2*^_*p*_ = 0.002. The mean differences between nose present and nose absent from the two groups were both negligible for the partially aware and unaware groups, − 0.308 vs 0.053 in raw FMS scores, suggesting that awareness had little if any effect.

### Navigation style

The experience of cybersickness could affect how subjects perform navigation. For example, we have informally observed that subjects often insert pauses in their movement as they start to feel sick. If active navigation is reduced in the more uncomfortable condition, it could potentially reduce the difference in cybersickness between conditions.

We tested whether there were any systematic differences between the navigation performance in the virtual nose conditions using three performance measures (see Methods). We found that navigation performance was comparable in the two virtual nose conditions. Multiple paired-sample t-tests did not reveal any significant difference in average translational speed (*t*(31) = 0.937, *p* = 0.356, *d*_*z*_ = 0.166), average rotation rate (*t*(31) = 1.221, *p* = 0.231, *d*_*z*_ = 0.216), or proportion of time without simulated movement, (*t*(31) = − 0.561, *p* = 0.579, *d*_*z*_ = − 0.099). For the last navigation metric of interest (proportion of time without simulated movement), we noticed that there was a violation of normality (*p* = 0.007), but the results remained qualitatively the same using the Wilcoxon signed-rank test (*p* = 0.832). Overall, there was no indication that navigation behavior was a confounding factor in our conditions.

### Presence and cybersickness

We first analyzed whether the virtual nose condition caused an increase in spatial presence, as measured by the SP subscale of the IPQ. We did not observe any significant difference in spatial presence between the nose-present (*M* = 3.475, *SD* = 0.760) and nose-absent (*M* = 3.394, *SD* = 1.040) conditions, *t*(31) = 0.598, *p* = 0.554, *d*_*z*_ = 0.106. No evidence was found suggesting a difference in spatial presence induced by the presence of the virtual nose.

We also analyzed individual differences in experienced motion sickness and reported spatial presence. No significant correlation was observed between the mean spatial presence scores across nose conditions and the mean rate of increase in FMS scores (*r*(30) = 0.028, *p* = 0.878). Overall, we did not find any evidence to support that spatial presence influenced the cybersickness increase rate for our experiment.

The Supplementary material has analyses of the other subscales and the total IPQ scores. The results were qualitatively the same across all IPQ subscales. No significant difference in the mean IPQ subscale scores was detected between the two virtual nose conditions, and no significant correlation between the individuals’ mean IPQ subscale scores and their mean rates of increase in FMS.

### Sex differences in cybersickness

We performed analyses to examine the sex differences in the virtual nose effect and experienced cybersickness.

To test whether the virtual nose had a different effect depending on sex, we conducted a 2 × 2 mixed ANOVA on the FMS scores using the virtual nose as a within-subjects factor and biological sex as a between-subjects factor. We found no interaction between sex and virtual nose (*F*(1, 30) = 0.058, *p* = 0.811, *η*^*2*^_*p*_ = 0.002). Analyses of the SSQ results showed a similar pattern, and are available in Supplementary material. There was no evidence that the virtual nose had a different effect on men and women.

To test for a sex difference in overall cybersickness, we performed an independent t-test comparing the mean rates of FMS increase for men and women. For this analysis, we used the rate of FMS increase rather than the difference in the last completed session (see Methods). Interestingly, although many previous studies have reported a sex difference in cybersickness, we did not observe any overall difference between the rates of increase in cybersickness (*t*(30) = 0.033, *p* = 0.974, *d* = 0.012).

## Discussion

Despite using a well-powered design, we did not observe any evidence that the virtual nose could be an effective countermeasure against cybersickness. Neither our primary (FMS) nor secondary (SSQ) measures revealed a significant difference between nose conditions, and the mean differences were close to zero. Even with the order effect controlled, the difference in cybersickness was not detectable. Results from our Bayesian analysis suggest that any missed effect of the virtual nose has an effect size | *d*_*z*_ |< 0.37, which is smaller than the effect size reported by Weinrich et al. (2022), *d* = − 0.52. We did not find any evidence that other potential confounders, such as navigation style and nose awareness, were different across the virtual nose conditions.

### Virtual nose may not be a useful rest frame

One explanation for our null finding is that a virtual nose does not provide a useful rest frame for resolving conflicts between visual and non-visual sensory cues in virtual reality.

Although previous studies have demonstrated the effectiveness of a visual rest frame in reducing cybersickness, the virtual nose offers fundamentally different feedback about self-motion compared to other rest frames (see Fig. 1). A background-based rest frame, such as a background grid or a cockpit, would distinguish between rotational motion induced by head movements and stimulated rotations. The virtual nose, which is a head-based reference frame, would not help in making such a distinction. For an augmented reference frame to serve as a reliable rest frame to improve cybersickness, it may be necessary that the implemented reference frame could reliably separate physically induced and stimulated rotational motion. This would explain why we did not observe a benefit from implementing a virtual nose.

Implementing a virtual nose as a reference frame is also arguably redundant, as it offers no additional feedback on self-motion that was not already present. For any VR display that attaches to the head, the screen boundaries would provide a head-based reference frame that provides equivalent information as a virtual nose. Although the HMD display frame has a different appearance than a human nose, it is still a visual reference frame that constantly moves with the user’s head. One subject even pointed out that the HMD frame already conveys the location of their physical nose, and that the addition of the virtual nose was unnecessary since they could already “feel their nose” without the simulated object. If the virtual nose could serve as a good rest frame, the HMD frame should theoretically be a good reference frame as well.

### Virtual nose may not facilitate the sense of spatial presence

We anticipated that the addition of a virtual nose might enhance the sense of presence inside the virtual world. However, the virtual nose did not appear to have a meaningful impact on spatial presence in our study.

It is possible that the virtual nose would have significantly improved spatial presence if it were positioned at a more natural location. In our experiment, because of how the HMD display was shaped, we were forced to position the virtual nose higher and further than where an actual nose would usually be located to enable its visibility. Some subjects commented that although they noticed there was clearly something present in the display bottom, they did not realize it was meant to resemble a human nose, as it felt too big and “away” compared to their own nose. If the virtual nose primarily reduces cybersickness through the facilitation of spatial presence, the unnatural positioning of the virtual nose may explain our failure to replicate the effect.

However, if an accurate depiction of a nose in the correct spatial location is essential to increase presence and reduce cybersickness, then this would not be a practical intervention. For most current HMDs, it is impossible to render an accurate depiction of the nose at the correct spatial location because it is outside the displays.

### Awareness of the virtual nose

Our debriefing results indicate that most subjects either did not notice the virtual nose or failed to recognize it as a virtual nose. After being told of the purpose of the experiment, most subjects were able to recall which experiment session had the virtual nose. Some subjects commented that they did notice a “blob” in the lower display during the navigation, but they did not realize it was meant to resemble a human nose. The lack of awareness of the virtual nose observed in our study was comparable to the results reported by Wienrich et al. (2022) for subjects who were not informed about the visual nose. In their subgroup of 10 subjects who received no hint about the virtual nose, only one explicitly reported the presence of the virtual nose, and three others detected something non-specific added to the display. As in our study, only a small proportion of subjects spontaneously noticed and recognized the virtual nose manipulation.

It remains possible that awareness and attention paid to the virtual nose contributed to our conflicting findings. One difference between ours and the previous study by Wienrich et al. (2022) was that they informed one group of subjects about the virtual nose, and encouraged them to attend to it throughout the virtual task. Furthermore, the virtual task used by Wienrich et al. (2022) might have encouraged more attention to the lower visual field, which would facilitate attention to and awareness of the nose. In their study, subjects performed a navigation task that required them to attend to where they would be landing on a platform, which may mandate attention to the display bottom, and subsequently the virtual nose itself. However, Wienrich et al. (2022) did not observe any significant difference in cybersickness between the nose awareness subgroups. The lack of difference between subgroups suggests that awareness was not a crucial factor, which argues against this explanation for our failure to replicate their results.

### Comparison to Weinrich et al. (2022)

The virtual environments and tasks used in our current study and Weinrich et al. (2022) were comparable. Despite using a similar manipulation of the virtual nose, similar complexity of the visual environments, and having comparable overall awareness of the virtual nose, we were unable to replicate the sickness-reducing effect of the virtual nose. However, there might be some other inconsistencies between the two studies which would explain our replication failure.

Compared to Weinrich et al. (2022), our study did not induce any vertically simulated movements (e.g., jumping). For our navigation task, subjects “walked” through a flat plane populated by various forest props. The task used by Weinrich et al. (2022) task required their subjects to “jump” from platform to platform occasionally, which may introduce a more severe sense of vertigo. However, there is no obvious reason that the virtual nose would be more effective at reducing vertigo specifically than other cybersickness symptoms.

Based on the FMS data, it appeared that our virtual task was more sickness-inducing than Weinrich et al. (2022). Our virtual navigation task produced an overall FMS score of 7.5, which is noticeably higher than the overall mean FMS score of 2.2 reported by Weinrich et al. (2022). It is possible that the virtual nose could only provide a benefit in conditions that cause minimal to low level of cybersickness. We believe that this is unlikely. Our sample consisted of individuals with varying susceptibility levels. Even if the virtual nose was only beneficial for people experiencing mild symptoms, we would still have expected some trend toward a benefit.

Another difference is that our study had more power to detect the effect of the virtual nose on cybersickness (if any). With a between-subjects design and only 30 total subjects (10 per group), Weinrich et al. (2022) only had 26% or 38% power to detect their reported reduction in the SSQ or FMS scores, respectively. Our current study used a within-subjects design with 32 subjects who performed in both conditions on separate days. Our design and sample size provided much higher statistical power (> 90%) to detect the hypothesized effect, greatly reducing the chance of an inflated estimate of effect size. If the original reported effect was an unfortunate false positive, this would explain our replication failure and the lower estimated effect size.

### The lack of sex difference in cybersickness

Our results did not reveal any detectable sex-based difference in cybersickness, either as measured by the FMS or the SSQ.

There remains debate about whether cybersickness depends on biological sex and previous findings are mixed. A number of studies have reported that the experienced symptoms tend to be more severe and manifest faster for females compared to the male population (e.g., Kim et al., 2021; Tian et al., 2022). Proposed explanations include differences in physiological mechanisms, previous VR/gaming exposure, or the female hormonal cycle (Arslanian-Engoren & Engoren, 2010; Golding et al., 2005). However, other studies have not found any sex differences in reported cybersickness (Melo et al., 2018; Petri et al., 2020), and a meta-analysis by Saredakis et al. (2020) concluded that there was no strong evidence for an effect of sex. If the previously reported effects are not reliable or due to some confounding factors, then the lack of sex difference in our study would not be surprising.

Stanney et al. (2020) have argued that the sex differences in cybersickness reported in previous studies can be largely attributed to poor fitting inter-interpupillary distance (IPD). Stanney et al. argue that, with the limited range adjustment for viewing IPD in most HMDs, females would tend to have more difficulty achieving good alignment compared to males, which could increase the risk of cybersickness. If non-fitting IPD is the main culprit behind the sex differences in cybersickness, this could explain the absence of such an effect in our study. In our study, we used a constant viewing IPD value (i.e., 62.5 mm) for all subjects, so males were as likely to have a non-fitting view IPD as females. However, Doty et al. (2023) found that IPD mismatch was related to recovery time from cybersickness and not severity of initial symptoms. Their results suggest that IPD mismatch would not explain the lack of sex differences in cybersickness in our study, which measured cybersickness during and immediately after the VR session.

## Conclusion

Using a well-powered sample, we were unable to replicate the finding that rendering a virtual reduces experienced cybersickness. Although other studies have demonstrated the effectiveness of a background-based rest frame, we argue that a head-based reference frame like the virtual nose would not be useful in overcoming the conflicts between visual and non-visual sensory systems. It remains possible that the virtual nose could have reduced sickness if it had sufficiently reduced stereoscopic field-of-view and/or appeared to be more realistic. However, such requirements would make the virtual nose a less practical countermeasure against cybersickness.

## Supplementary Information

Supplementary material 1.

## Data Availability

The data and materials are available at the public repository: https://osf.io/dyma4. Preregistration: The experiment was preregistered on the Open Sciences Framework prior to data collection: https://osf.io/zcaes.
