# Supplementary material for: Preregistered test of whether a virtual nose reduces cybersickness
Source: Cogn Res Princ Implic. 2024 Oct 29;9:74. doi: 10.1186/s41235-024-00593-3 (PMC11522211; doi:10.1186/s41235-024-00593-3)
Supplement: Supplementary file 1 — Supplementary material 1. [file 41235_2024_593_MOESM1_ESM.docx]

# Supplementary Material

Table 1. Differences in the Mean IPQ Subscale Scores Between the Nose Conditions

|  | Nose-Present | | Nose-Absent | |  |  |  |  |
| --- | --- | --- | --- | --- | --- | --- | --- | --- |
|  | *M* | *SD* | *M* | *SD* | *t* | *df* | *p* | *d_z_* |
| IPQ Total | 2.781 | .690 | 2.652 | .829 | 1.242 | 31 | .224 | .220 |
| IPQ INV | 2.648 | 1.179 | 2.461 | 1.102 | .917 | 31 | .366 | .162 |
| IPQ REAL | 1.836 | .939 | 1.727 | .802 | 1.045 | 31 | .304 | .185 |

Table 2. Correlations Between IPQ Subscale Scores and Mean Rates of Increase in FMS

|  | Correlation with the Mean Rates of Increase in FMS (*r*) | *p* |
| --- | --- | --- |
| IPQ Total | .051 | .783 |
| IPQ INV | .066 | .719 |
| IPQ REAL | .048 | .793 |

Table 3. ANOVA Results of “Biological Sex” X “Virtual Nose” on SSQ-Total

|  | *SS* | *df* | *MS* | *F* | *p* | *η^2^_p_* |
| --- | --- | --- | --- | --- | --- | --- |
| Between-subjects effects |  |  |  |  |  |  |
| Biological Sex | .041 | 1 | .041 | .151 | .701 | .005 |
| Error | 8.200 | 30 | .273 |  |  |  |
| Within-subjects effects |  |  |  |  |  |  |
| Virtual Nose | <.001 | 1 | <.001 | <.001 | .993 | <.001 |
| Nose * Sex | .009 | 1 | .009 | .195 | .662 | .006 |
| Error | 1.319 | 30 | .044 |  |  |  |

Table 4. ANOVA Results of “Biological Sex” * “Virtual Nose” on SSQ-Nausea

|  | *SS* | *df* | *MS* | *F* | *p* | *η^2^_p_* |
| --- | --- | --- | --- | --- | --- | --- |
| Between-subjects effects |  |  |  |  |  |  |
| Biological Sex | .009 | 1 | .009 | .027 | .870 | .001 |
| Error | 9.978 | 30 | .333 |  |  |  |
| Within-subjects effects |  |  |  |  |  |  |
| Virtual Nose | .020 | 1 | .020 | .425 | .520 | .014 |
| Nose * Sex | .001 | 1 | .001 | .012 | .914 | <.001 |
| Error | 1.389 | 30 | .046 |  |  |  |

Table 5. ANOVA Results of “Biological Sex” * “Virtual Nose” on SSQ-Oculomotor_Distrub

|  | *SS* | *df* | *MS* | *F* | *p* | *η^2^_p_* |
| --- | --- | --- | --- | --- | --- | --- |
| Between-subjects effects |  |  |  |  |  |  |
| Biological Sex | .108 | 1 | .108 | .704 | .408 | .023 |
| Error | 4.617 | 30 | .154 |  |  |  |
| Within-subjects effects |  |  |  |  |  |  |
| Virtual Nose | .043 | 1 | .043 | 1.098 | .303 | .035 |
| Nose * Sex | .025 | 1 | .025 | .654 | .425 | .021 |
| Error | 1.163 | 30 | .039 |  |  |  |

Table 6. ANOVA Results of “Biological Sex” * “Virtual Nose” on SSQ-Disorientation

|  | *SS* | *df* | *MS* | *F* | *p* | *η^2^_p_* |
| --- | --- | --- | --- | --- | --- | --- |
| Between-subjects effects |  |  |  |  |  |  |
| Biological Sex | .032 | 1 | .032 | .092 | .763 | .003 |
| Error | 10.411 | 30 | .347 |  |  |  |
| Within-subjects effects |  |  |  |  |  |  |
| Virtual Nose | .010 | 1 | .010 | .187 | .668 | .006 |
| Nose * Sex | <.001 | 1 | <.001 | .005 | .943 | <.001 |
| Error | 1.666 | 30 | .056 |  |  |  |
